# Epigallocatechin Gallate: The Emerging Wound Healing Potential of Multifunctional Biomaterials for Future Precision Medicine Treatment Strategies

**DOI:** 10.3390/polym13213656

**Published:** 2021-10-23

**Authors:** Mazlan Zawani, Mh Busra Fauzi

**Affiliations:** Centre for Tissue Engineering & Regenerative Medicine, Faculty of Medicine, Universiti Kebangsaan Malaysia, Kuala Lumpur 56000, Malaysia; P109645@siswa.ukm.edu.my

**Keywords:** epigallocatechin gallate, chronic skin wound, antioxidants, tissue engineering, biomaterials, reactive oxygen species, diabetic foot ulcer

## Abstract

Immediate treatment for cutaneous injuries is a realistic approach to improve the healing rate and minimise the risk of complications. Multifunctional biomaterials have been proven to be a potential strategy for chronic skin wound management, especially for future advancements in precision medicine. Hence, antioxidant incorporated biomaterials play a vital role in the new era of tissue engineering. A bibliographic investigation was conducted on articles focusing on in vitro, in vivo, and clinical studies that evaluate the effect and the antioxidants mechanism exerted by epigallocatechin gallate (EGCG) in wound healing and its ability to act as reactive oxygen species (ROS) scavengers. Over the years, EGCG has been proven to be a potent antioxidant efficient for wound healing purposes. Therefore, several novel studies were included in this article to shed light on EGCG incorporated biomaterials over five years of research. However, the related papers under this review’s scope are limited in number. All the studies showed that biomaterials with scavenging ability have a great potential to combat chronic wounds and assist the wound healing process against oxidative damage. However, the promising concept has faced challenges extending beyond the trial phase, whereby the implementation of these biomaterials, when exposed to an oxidative stress environment, may disrupt cell proliferation and tissue regeneration after transplantation. Therefore, thorough research should be executed to ensure a successful therapy.

## 1. Introduction

The split skin graft (SSG) and commercially available tissue engineering medical products (TEMPs) are the gold standard treatments for skin injuries. However, some complications are commonly highlighted, which are high susceptibility to trauma and protracted wound care for both the donor and beneficiary [1]. Hence, venturing into immediate treatment for cutaneous injury is a realistic approach for improving the healing rate and minimising the risk of complications that could delay normal wound healing mechanisms. Functionalised biomaterials have been proven to be a potential strategy for skin wound management; hence, antioxidant incorporated biomaterials play a vital role in the new era of functional biomaterials in tissue engineering [2]. ROS plays a crucial role in the wound healing process, in which a balanced level of ROS is essential, particularly in combatting delayed cutaneous injury caused by chronic conditions such as diabetes mellitus or peripheral vascular disease.

A cutaneous wound is defined as an injury that occurs within the skin, connective tissues, or the mucus membrane, resulting in the organs’ structural and/or functional defects [3]. A wound can be categorised into two main types: acute and chronic wounds. Chronic wounds demonstrate stalled inflammatory phases resulting in biofilm development, bacterial clusters, and protease elevation at the wound site. The domination of protease above the inhibitors leads to extracellular matrix destruction, thus accelerating the proliferation and inflammation phases. This process then triggers ROS to rise, resulting in premature cells and defective extracellular matrix proteins [4].

Prior to pathological injuries, the damaged tissue undergoes a complex healing process to self-repair. During this process, several growth factors, cells, free radicals, and pro-oxidant species are engendered by the body’s immune response. In small concentrations, the pro-oxidant species aid in various physiological facets [5]; however, gradually over spatiotemporal factors, the overflow of free radicals and pro-oxidant species on the wound site may be vital. Therefore, various studies have propounded incorporating an antioxidant and/or free radical scavenging function in biomaterials to aid tissue regeneration and body function repair [6].

The state-of-the-art development of these functionalised biomaterials is to transpose pure antioxidants with ROS-inducing particles that can modulate oxidation-reduction mediated cell homeostasis and successfully attain redox equanimity for in vivo as well as 3D in vitro cell cultures for future clinical implantation.

## 2. Wound Healing Phases

**Figure 1:**
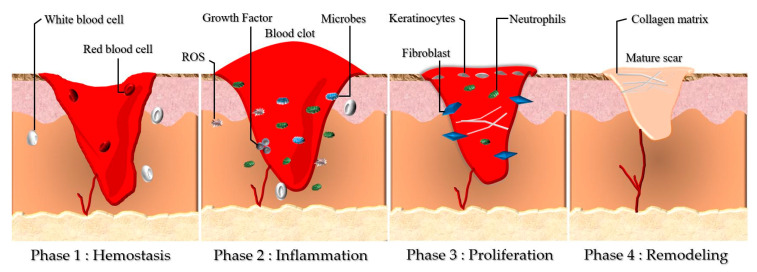
Cutaneous wound healing myriad includes four continuous phases, which involve Phase 1: Hemostasis, Phase 2: Inflammation, Phase: 3 Proliferation, and Phase 4: Remodelling. During these four phases, the blood platelets are activated before the injury, forming a blood clot, which plays a role in leukocyte recruitment. Then, as shown in Phase 2, neutrophils and macrophages remove debrides and fight against infection (bacteria, dead cells, pathogens). The angiogenesis process is initiated in Phase 3, where fibroblasts would migrate and proliferate. The collagen matrix is formed to restore the extracellular matrix in the remodelling phase (Phase 4), which turns into granulation tissue (mature scar).

The contemporary perception of the wound healing process is centred on the myriad of phases and the involvement of the signalling factors. Wound healing is a complex dynamic process that involves four distinct stages: (1) hemostasis, (2) inflammation, (3) proliferation and migration, and (4) remodelling, as shown in Figure 1.

### 2.1. Phase 1: Hemostasis

Hemostasis is the first challenge of cell repair. During this phase, the platelets are activated, aggregate, and adhere to the damaged and defective squamous endothelial to conserve hemostasis via coagulation. As the phenomena are introduced, fibrin from the fibrinogen forms an embolus that acts as an impermanent extracellular matrix (ECM). The activated cells (platelets, neutrophils, and monocytes) release several proteins and growth factors, for example the transforming growth factor β (TGF-β) and platelet-derived growth factor (PDGF). An alteration of the hemostasis phase was observed in diabetes mellitus (DM) patients by reducing the hyper-coagulation and fibrinolysis [7].

### 2.2. Phase 2: Inflammation

This phase is characterised by neutrophils, mast cells, and macrophages causing the production of inflammatory cytokines (interleukin 1 (IL-1), tumour necrosis factor-alpha (TNF-α), interleukin 6 (IL-6), and interferon-gamma (IFN-γ)) as well as the growth factors of PDGF, TGF-β, insulin-like growth factor 1 (IGF-1), and epidermal growth factor (EGF) as the main essentials in the wound healing process [8]. Xiao et al. reported the presence of a cytokine imbalance in diabetic patients, which could alter the wound healing process. The modified cytokine distribution pattern led to the reduction of their function, causing the wound to be prone to infection [7].

### 2.3. Phase 3: Proliferation

The migration and proliferation processes begin in this phase, including wound contraction to angiogenesis action. These actions include restoring oxygen supply; the creation of ECM proteins, vitronectin, and collagen; and the proliferation and migration of fibroblasts and keratinocytes, which are essential for integrity recovery and functionality of the tissues [8]. Hyperglycaemic conditions in DM patients alters the ability of the fibroblasts and keratinocytes to migrate and proliferate. Therefore, the abnormal cells cause the stagnation of angiogenesis, which eventually affects the healing process [9].

### 2.4. Phase 4: Remodelling

The remodelling phase is in action seven days after the injury and can last up to 6 months. Collagen III is synthesised and replaced with collagen I to restore the ECM. The wound becomes resistant, and the mature scar tissue is formed (granulation tissue) [7,10]. Alteration of the fibroblasts’ functionality in diabetics patients causes the deformation of the wound closure. In [10], Maione et al. stated that the unresponsive action might be due to the TGF-β and abnormal ECM production.

The epithelial wound healing phases are deleterious to the healthy cells’ proliferation process, whereby the ROS is crucial for the wound healing activity at the basal level. Various studies have highlighted the significance of a balance in ROS for wound healing because a total suppression of these free radicals and an excessive number of oxidants could impair wound healing. Furthermore, ROS have been implicated as essential cell signalling mediators in wound repair. However, the disproportionate production of these free radicals may be harmful [11].

## 3. Oxidative Stress in Chronic Wounds

The intricate equilibrium of ROS and their pro-oxidants are crucial in wound healing as ROS is essential to initiate wound repair. Lipid peroxidation, protein, and DNA alteration-mediated oxidative stress cause augmented cell apoptosis leading to wound healing impairment [12]. Physiologically, neutrophils and macrophage-mediated NADPH oxidases (NOX) generate low levels of ROS, which are responsible for respiratory ruptures all through phagocytosis of the inflammatory phase. On the contrary, in chronic wound conditions, NOX activation is intensified, leading to excessive ROS production and thus hastening the inflammatory phase and oxidative stress cellular damage [13,14,15].

**Figure 2:**
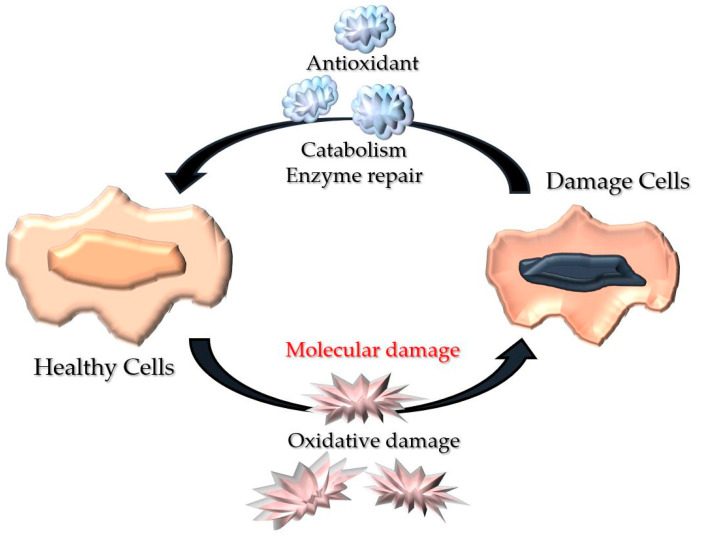
Representative illustration of wound oxidative stress. Healthy cells recuperate via metabolism amelioration and enzyme repair before oxidative mutilation to circumvent apoptosis or impairment.

ROS is a small oxygen-derived molecule mainly produced by the respiratory chain in the mitochondria. They are oxidising agents and significant contributors to cell damage [16], but they also have beneficial roles in preparing regular wound healing responses [9]. Therefore, a suitable balance between low and high levels of ROS is essential. Low levels of ROS are beneficial in protecting tissues against infection and stimulating effective wound healing [10]. However, when in excess, ROS produce oxidative stress leading to cell damage and a pro-inflammatory status [17]. Redox imbalance occurs when the levels of ROS exceed the capacity of endogenous antioxidants to scavenge them, which dysregulates the healing process [5,18] (Figure 2).

## 4. Significance of Antioxidants in Chronic Wound Healing

The oxygen molecule is made up of two electrons with identical spin quantum numbers, from which ROS are derived. These ROS are chemicals that contain oxygen that is highly reactive compared to the ground state oxygen [19]. Hydroxyl radicals (OH) or superoxide anion radicals (O_2_) are ROS species that are generated through the oxygen to water conversion in human cells. When there is an imbalance in the number of free radicals and antioxidants, oxidative stress-related diseases will emerge, leading to complications such as chronic wound healing [19,20]. Therefore, this imbalance needs to be overcome with radical scavenging molecules.

Antioxidants are molecules that avert oxidative occurrence. These compounds detoxify ROS to obviate damage effects via the multi-mechanism of free radical scavengers and the inhibition of lipid peroxidation. These mechanisms aid in potentiating the immune mechanism during a particular septic state or even ageing. Antioxidants preserve and stimulate the function of immune cells against homeostatic disturbances [21,22].

**Figure 3:**
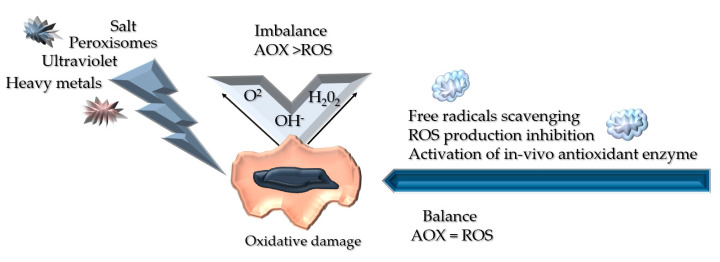
Illustrates the reactive oxygen species (ROS) generation, impact, and its eradication therapy. Oxidative stress can be triggered by external stimuli such as UV and salt, which will interfere with the wound’s homeostasis, leading to vital damage towards the cell’s continuous development. However, in a balanced state, these reactive oxygen species can be inhibited by antioxidants via free radical scavenging hence aiding in antioxidant enzyme system activation, which endorses non-enzymatic antioxidants (AOX) [19].

ROS and its corresponding pro-inflammatory cell signalling hold a vital role in wound healing [23,24] (Figure 3). Exogenic antioxidants are emitted as soon as the overflow of oxidative stress coerces the endogenic antioxidants, permitting inhibition of the inflammatory pathway that leads to the acceleration of wound healing due to the ROS balance [25].

There are two types of cutaneous antioxidants: enzymatic and non-enzymatic. Enzymatic antioxidants are endogenic molecules that are originated from the mechanism of oxidative cells, catalase, superoxide dismutase, and glutathione peroxidase [26], whereas non-enzymatic antioxidants are equally endogenic and exogenic, and are commonly attained from phytoconstituents classified under polyphenols and carotenoids [26,27]. Polyphenols and carotenoids are widely used in wound healing management for their anti-inflammatory, antibacterial, and antioxidant properties [28,29]. Furthermore, polyphenols and carotenoids’ ability to sustain inflammatory signalling have shed light on new chronic wound healing therapies. Both polyphenols and carotenoids have also been proven to play a vital part in wound healing phases including inflammation, proliferation, and remodelling [27].

ROS play a vital role in the body’s physiological process. However, they bring more harm than good as they are responsible for hastening ageing in organisms and deteriorating food intake. Various studies have shown that these free radicals not only cause ageing and impairment, but are also one of the primary sources of degenerative disease [30,31,32]. The most common representation of ROS damage can be seen in chronic wound healing. The free radicals are highly reactive, which help initiate the signalling pathway of the immune response to stimulate redox-mediated intracellular oxidation and bacterial resistance [33].

However, in a substantial amount, the ROS will lead to oxidative stress towards nucleic acids, proteins, and lipids. Oxidative stress may lead to cell apoptosis and systemic injury, which then cause wound-healing impairment. On the other hand, antioxidants have been proven to efficiently restore metabolic and enzymatic repair for cell recovery [34,35]. Therefore, antioxidant incorporated biomaterials are in great demand in the tissue engineering field, as the development of high-efficiency novel bioscaffolds with outstanding biocompatibility and capability to sustain intracellular redox balance is anticipated to overcome metabolic disorder induced by oxidative stress [19].

## 5. Chronic Wounds and Their Complications

There are a number of chronic wound aetiologies burdening the healthcare institution. Patients with chronic diseases such as diabetes and obesity are the two major contributors to the increase in chronic wounds worldwide [36]. This type of wound is commonly associated with comorbidities, making it difficult to trace it as a disease.

Diabetes mellitus (DM) is a chronic disease that occurs as the result of the reduction of the insulin hormone in the body or when the body itself is not able to utilise insulin effectively. Insulin, a hormone secreted from the pancreas, assists the regulation of blood glucose in the body. DM is also classified as a metabolic disease and is 1 of 4 priorities for non-communicable diseases, which have the highest impact on health and the socioeconomic burden worldwide [37]. The high prevalence of diabetes in adults increases the risk of foot problems mainly due to neuropathy and peripheral arterial disease [38]. DFU is commonly present from the distal level to the ankle among diabetic patients [39], in which diabetic peripheral neuropathy affects up to 50% of diabetic patients without exhibiting any symptoms [7]. Each year, approximately one million amputations are performed on diabetic patients worldwide [40,41]. DFU requires special care and coordination, ideally from a multidisciplinary foot care team.

Diabetic foot diseases (DFD) are the result of uncontrolled diabetes and are the main complications that occur in DM patients contributing to neuropathy, peripheral vascular disease, high foot plantar pressure, foot trauma, atherosclerosis, and thus, an increase in morbidity [41,42,43]. The combination of neuropathy or peripheral vascular disease will eventually lead to the development of a diabetic ulcer. DFU frequently involves full-thickness skin loss, which deteriorates the bone, joint, and soft tissues [42]. These will inflict complications on diabetic patients; hence, amputations are performed in >50% of patients. The wound healing phase is mainly compromised during this complication, exposing the ulcers to infections, stagnant proliferations, a stalled inflammatory phase, prolonged angiogenesis, and impaired foot deformity. These events will eventually lead to surgical debridement and amputation of the affected area to obviate further systemic sepsis [44,45].

## 6. Epigallocatechin Gallate (EGCG)

**Figure 4:**
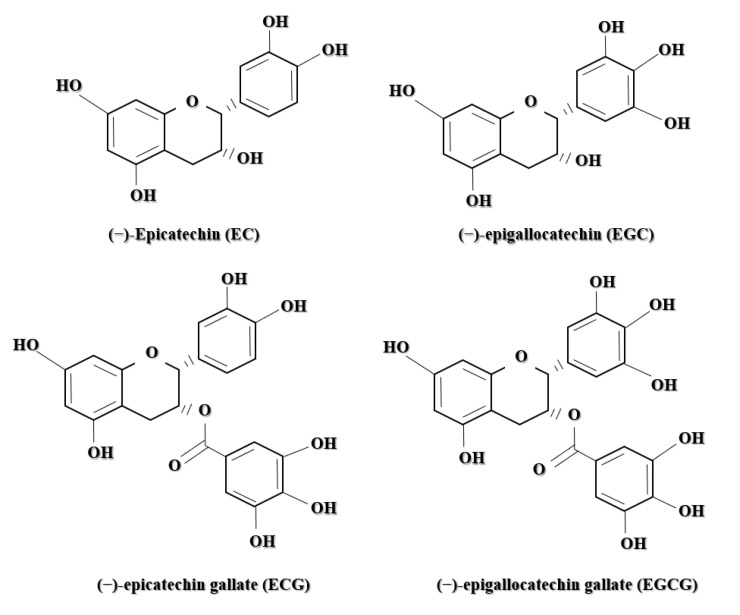
The chemical structure of four (4) primary polyphenols in *Camelia sinensis*: epicatechin (EC), epigallocatechin (EGC), epicatechin gallate (ECG), and epigallocatechin gallate (EGCG).

*Camellia sinensis* possesses several phenolic compounds consisting of three main groups: flavones, flavanols, and flavonols [8]. Each compound has a unique heterocyclic C-ring, which distinguishes it from its primary structure as shown in Figure 3. The most abundant compound of flavanols in green tea comprises approximately one-third of the dry tea leaf. Flavanols are primarily distributed into four major molecules: epicatechin (EC), epicatechin gallate (ECG), epigallocatechin (EGC), and EGCG [3] as shown in Figure 4.

### 6.1. Chemical Structure of Epigallocatechin Gallate (EGCG)

**Figure 5:**
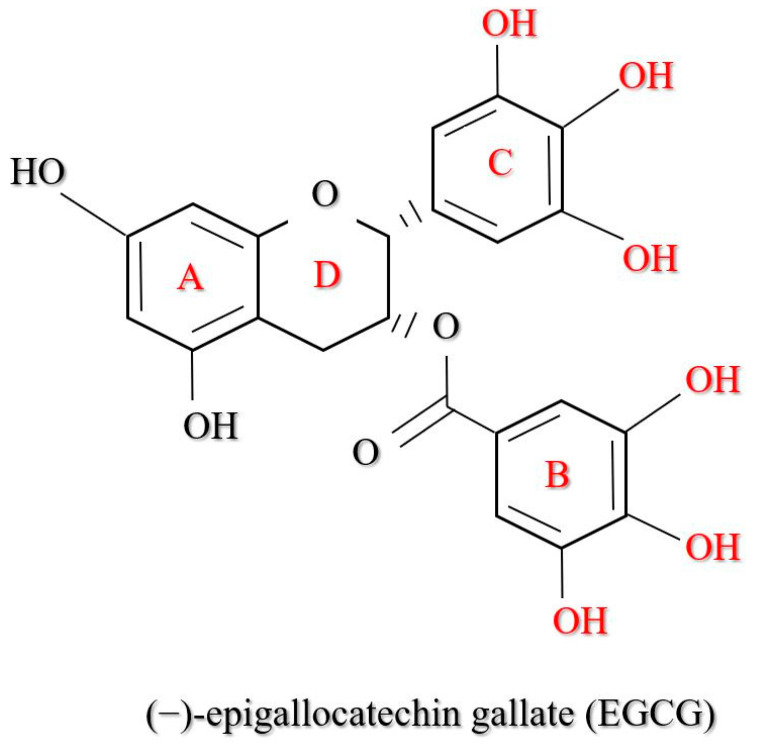
The EGCG comprises four rings designated as A, B, C, and D. The A and C rings form a benzopyran ring system, which relates to the pyrogallol (the B ring) and gallate (the D ring) moiety at the C-2 and C-3 positions, respectively. The role of the functional groups in the antioxidant properties of EGCG was described based on other studies.

With a total average of 65% catechin content, EGCG is the main contributor to most of the therapeutic phenomena exerted by green tea (Figure 5). The flavanol catechin EGCG consists of three hydroxyphenyl and hydroxybenzoate moieties [46] with properties of down-regulating inflammatory pathways used in the production of cosmetics and dermatology [47]. In addition, not only is EGCG known for its antioxidant activity, but the green tea catechin is also widely exploited for its anticarcinogenic properties [38], anti-ageing efficiency in the nutraceutical fields [39], photo-protection (as a photo carcinogenesis inhibitor) [36,40], and neuroprotective effect [37], as it plays an active role against β-amyloid aggregations.

### 6.2. Potential Wound Healing Mechanism of EGCG

EGCG inhibits the signalling cascade of PDGF and EGF via binding to their receptors during the inflammatory phase in a perpetual manner. Furthermore, EGCG suppresses the expression of the EGF receptor, interrupting the epithelialisation process. In the inflammation phase, inflammatory immune cells of mast cells, neutrophils, and macrophage-mediated free radicals, cytokines, and growth factors are produced. Meanwhile, the elevation of micro vascularity transports immune cells, oxygen, and nutrients to the wound site as macrophages yield PDGF, fibroblast growth factors, TGF-β1, and vascular endothelial growth factor, with EGCG continuously suppressing the PDGF receptor [27].

**Figure 6:**
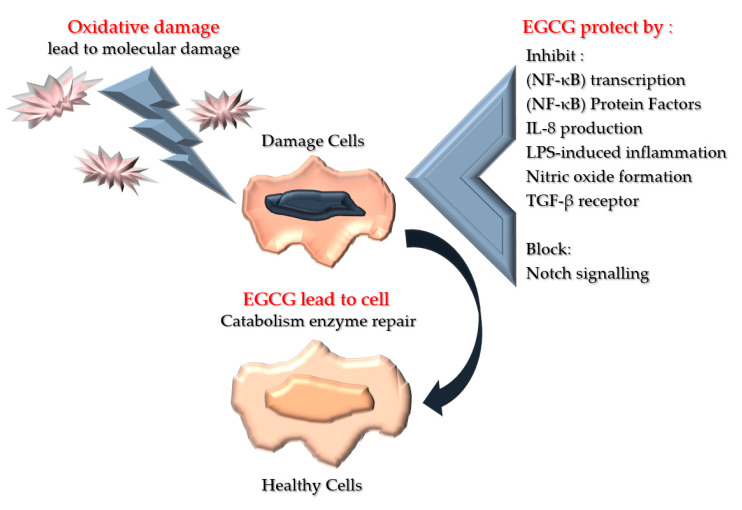
Illustration of the mechanism of EGCG towards molecular damage. This molecular damage will lead to vital damage towards the cell’s continuous development, and hence cell apoptosis. However, the antioxidant properties of EGCG are able to overcome oxidative damage and lead to cell recovery and enzyme repair.

Additionally, EGCG suppresses the IL-8 production, hence diminishing neutrophil aggregation that leads to the inhibition of the inflammatory response and the formation of ROS enzymes such as cyclooxygenase, lipoxygenase, and xanthine oxidase, affecting nitric oxide production via the nitric oxide synthase interface. Moreover, EGCG can also stimulate free radical detoxification enzymes, which lead to a rapid wound healing process [48]. EGCG’s functionality as an antioxidant is via the inhibition of nitric oxide production, which suppresses free radical production and balances the wound environment. Remarkably, EGCG also plays a vital role in shielding the endothelial cells in the vascular system [49] (Table 1 and Figure 6).

Along with their known antioxidant activity, accumulating evidence has shown the antimicrobial potential of EGCG towards Gram-negative bacteria (*E. coli*, *Pseudomonas*, *Salmonella*) and Gram-positive bacteria (*Staphylococcus aureus*, *Bacillus*) with 95% zone inhibition [54]. This is a crucial discovery as EGCG can be utilised as the gold standard to enhance healing recovery for the management of DFU as chronic wounds mainly face stalled inflammatory phase caused by the overproduction of ROS and biofilm microbial infection. A study conducted by Hassan et al. showed a prevalence of 77.3% monomicrobial and 22.7% polymicrobial infections, respectively, in diabetic foot ulcers identified as *A. baumanni*, *S. aureus*, *K. pneumonia*, and *S. aureus* [55]. Furthermore, an in vivo study on chronic plantar showed a significant result as the wound healed drastically (>84% ulcer reduction in 10 days) with the application of topical EGCG [54].

Studies that exploit the effectiveness of EGCG towards DFU patients and diabetic induced in vivo models have discovered enhanced neovascularisation, increased collagen, granulation tissue thickness, a rise of capillary density, enhanced angiogenesis, and cellular reorganisation [51,53,56,57,58]. Hence, the antibacterial and free radical scavenging ability of EGCG suggests effective therapeutic modality for dermal wound management.

## 7. Advancement in Tissue Engineering

Tissue engineering is an interdisciplinary field integrating principles of regenerative medicine and biology to develop biological substitutes that aid in repairing and reinstating organ function [59]. Tissue engineering plays a significant role in regenerative medicine, which holds the ideologies of biocompatible, bio-mimic materials science and engineering to develop native tissue substitutes in order to restore and maintain the homeostasis of damaged skin. In this era, tissue engineering approaches mainly focus on two major categories: tissue-derived biomaterials and scaffold-based therapy. The conventional tissue-derived or cell-based therapy utilises the convenience of coalescing cells with natural or synthetic polymers that are bioinert. The scaffold-based therapy is mainly based on the host’s ability to regenerate, hence suitably aligning the direction of cell proliferation and regeneration [60].

With technological advancements, other series of therapies for chronic wound management have been implemented, such as the development of skin substitutes, negative pressure wound therapy, hyperbaric oxygen, fabrication of novel wound dressings that implicate growth factors, and the use of tissue-derived biomaterials. In tissue engineering, biomaterials play a vital role as a provisional bioscaffold for tissue repair and regeneration [2,61,62,63].

### 7.1. Tissue-Derived Biomaterials

Tissue-derived biomaterials utilise both tissue engineering and regenerative medicine to accelerate and assist chronic wound healing. As it is derived from tissue, these biomaterials mimic the native human soft tissues, hence nurturing a biocompatible microenvironment [64]. These biomaterials are harvested from cadaveric tissue allografts, placenta, submucosa of the small intestines, and algae [64,65]. The epidermis of the cadaveric human skin is removed to fabricate or construct the cadaveric allograft to avoid biological rejection [65]. Other sources of these materials are human amnion and chorion membranes (dehydrated), the layer of epithelial cells, basement membranes, and the avascular connective tissue matrix, which have been scientifically proven to accelerate wound healing and showed elevated wound closure compared to the standard wound management. These biomaterials are excellent in mimicking the ECM scaffolds, promoting high biocompatibility for wound healing purposes.

### 7.2. Hydrogel-Based Biomaterials

Engineered hydrogels have the advantage of being able to alter their properties and have more defined ingredients compared to tissue-derived matrices. They are usually designed and engineered to mimic the ECM found in the natural soft tissues [64]. These biomaterials can be made into high-water-content hydrogels, sponge and patch structures, or other architectures. They can also be crosslinked, dehydrated, freeze-dried, or electrospun [66,67]. Poly-N-acetyl glucosamine (pGlcNAc) is a microalgae-derived matrix that has been approved by the Food and Drug Administration (FDA) for the treatment of DFU [68]. However, the most favoured material in the development and fabrication of hydrogel for DFU management is fibrin. Fibrin has been proven to accelerate angiogenesis and regulate the wound inflammation process, thus facilitating wound healing [69].

## 8. Epigallocatechin Gallate (EGCG) Treatments for Wound Healing

Epigallocatechin gallate has been utilised to assist stall inflammation phase and accelerate chronic wound healing, mainly for its antioxidant properties. Throughout the years, many successful studies have been conducted in tissue engineering, medicine, and the pharmaceutical field by incorporating this potent green tea catechin (Table 2).

### 8.1. Epigallocatechin Gallate (EGCG) Incorporated Biomaterials

In a recent study, Lee et al. evaluated the antioxidative and ROS scavenging properties of EGCG-coated biomaterials designed for tissue engineering. They found that EGCG-coated polycaprolactone (PCL) film increases cell attachment, viability, and proliferation of human adipose-derived stem cells (hADSCs) against H_2_O_2_ exposure while regulating cell signalling that diminishes apoptotic genes, thus augmenting the expression of the anti-oxidative enzyme. The amalgamated EGCG-coated PLLA fibre spheroids showed better cell viability and anti-oxidative activities in response to H_2_O_2_ induced oxidative stress compared to the control [53].

Recently, self-healing hydrogel has been widely explored due to its outstanding mechanical strength and flexibility. Through a novel finding, Zhao et al. effectively developed a facile multifunctional hydrogel dressing with self-healing ability. The green tea-derived hydrogel showed a highly biocompatible and flexible mechanical strength that effectively exerted anti-oxidative properties via ROS assay and aided in wound healing with a 2% higher healing rate and neovascularisation in diabetic induced in vivo models, hence facilitating the proangiogenic properties [70].

In another successful study, Kar et al. defined the potential of a bio-inspired hydrogel nanocomposite system as a productive wound dressing material with multi-beneficial components for rapid anti-scarring wound healing, that is, NeuSkin-F^®^, a commercially marketed collagen film bioscaffold product [71]. They observed that EGCG showed fast wound closure through synergistic interaction without any inherent toxicity throughout the study.

Sun and his team [72] created a novel nanoparticle in the hopes of aiding in the acceleration of chronic wound healing by incorporating various bioinert materials such as EGCG, ascorbic acid, gelatine, and chitosan to ensure cell biocompatibility. These nanoparticles are named EV NPS and this novel material has successfully shown better performance in vivo by completely healing a full-thickness skin wound within 10 days with thicker epithelium, abundant neovascular vessels, hair follicles, and other skin attachments compared to the control.

**Figure 7:**
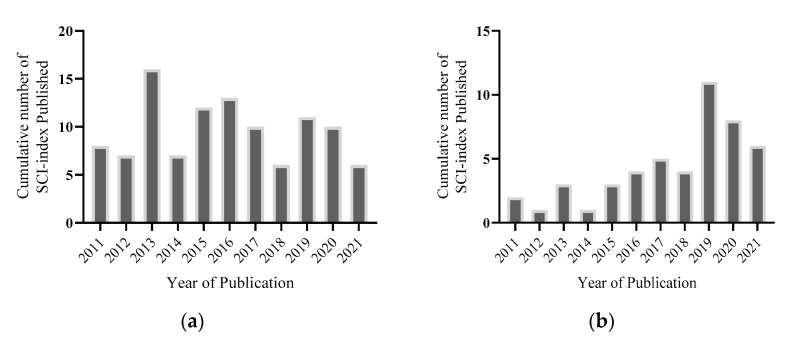
(**a**) Illustrated cumulation of SCI-indexed publications on Web of Science demonstrating research interest of EGCG in tissue engineering with the search terms of “antioxidants”, “skin wound”, “reactive oxygen species”, “epigallocatechin gallate”, “biomaterials”, “chronic wound healing”, “mechanism of actions”, and “tissue engineering”. (**b**) Graph signifies the publications referencing “EGCG” and “biomaterials” for skin wound healing over 10 years.

Meanwhile, a study by Pires et al. utilising a nanofiber membrane consisting of polycaprolactone and a gelatine carrier discovered that EGCG-loaded nanofibers could halt oxidative-stress cell damage. This finding showed great potential for wound healing and skin tissue engineering [57]. Publications on EGCG biomaterials over the past 10 years, the numbers of which are shown in Figure 7, demonstrate less exploration of the synergistic effect on EGCG ability incorporated with biomaterials in wound healing applications.

### 8.2. Epigallocatechin Gallate (EGCG) Incorporated Treatment

Several studies have incorporated EGCG as a topical application for use in chronic wound healing (Table 3). Huang et al. concluded that EGCG could improve wound healing by targeting the Notch signalling in STZ-induced diabetic mice, hence proving that EGCG accelerates wound healing under the condition of diabetes [51]. The study was successful as they retained positive results through H&E staining, which shows EGCG accelerates chronic wound healing during the inflammation phase, and the impairment completely healed after day 8 compared to the untreated group. However, more research should be carried out to further demonstrate the mechanism of the interaction in vivo.

In another study by Prakoeswa et al., 1% EGCG ointment showed an acceleration of wound closure in the treatment (1% EGCG Eucerin ointment) group compared to the control group by applying the ointment using Framycetin Gauze Dressing (FGD) containing the antibiotic. The study obtained great information supported by other previous studies, stating that EGCG inhibits the transcription factors and protein activators of NF-κB. These inhibitions reduced the production of inflammatory factors that aid in healing chronic wounds after the inflammation phase [54].

## 9. Data Extraction Management

A bibliographic study was carried out until the end of August 2021 in which the data and information of this review were obtained from multiple databases and search engines such as Cambridge Media, Elsevier, Wiley, Sage, MDPI, IEEE, Springer Nature, Oxford University Press, and American Scientific Publishers. All studies addressing EGCG as a wound healing mechanism in vivo, in vitro, or in clinical studies, and review articles with the result or conclusion of antioxidants, wound healing properties, or ROS inducement and changes in the released growth factor were included. The search terms including “antioxidants”, “skin wound”, “reactive oxygen species”, “epigallocatechin gallate”, “biomaterials”, “chronic wound healing”, “mechanism of actions”, and “tissue engineering” were used during the bibliographic investigation. There are limiting factors on the total related papers on EGCG under this review scope from the search.

## 10. Conclusion and Future Perspectives

EGCG has shown promising antioxidant and free radical scavenging properties, which are proven through various studies. Various mechanisms can achieve the wound healing properties of EGCG, including targeting Notch, inhibiting nuclear factor-kappa B (NF-κB) transcription, inhibiting (NF-κB) protein factors, IL-8 production, LPS-induced inflammation, nitric oxide formation, ROS enzymes, and the activation of SOD. Hence, ECGC is an intriguing component to be utilised in tissue engineering. Currently, the immediate treatment of cutaneous injuries is a realistic approach to improve the rate of healing and minimise the risk of complications. Functionalised biomaterials have been proven to be a potential strategy for use on chronic skin wound management. Hence, the development of novel EGCG incorporated biomaterial is actively being studied to achieve the optimum bioscaffold for chronic wound healing management.

**Figure 8:**
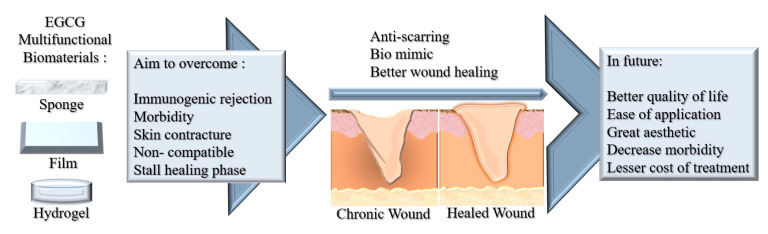
Schematic figure of the future perspectives on the development of EGCG multifunctional biomaterials.

Tissue engineering plays a major role in regenerative medicine that holds biocompatible, bio-mimic materials science and engineering ideologies to develop native tissue substitutes. The advancement of tissue engineering towards combating chronic wound healing has created a number of state-of-the-art biomaterials that help increase patient quality of life, hence reducing the cost of treatment and morbidity (Figure 8). However, a thorough understanding of the clinical challenges of combining these biomaterials is crucial for developing novel functionalised tissue engineering bioscaffolds.

## Figures and Tables

**Table 1:** Epigallocatechin gallate (EGCG) wound healing potential.

Wound Healing Potential	Mechanism
Anti-Inflammatory	Inhibit nuclear factor-kappa B (NF-κB) transcription [12,27,30]
	Inhibit (NF-κB) protein factors [12,30]
	Inhibit IL-8 production [50]
	Inhibit LPS-induced inflammation [51,52]
	Block Notch signaling [51]
Antioxidant	Inhibit nitric oxide formation [19]
	Inhibit ROS enzymes [53]
	Activation of SOD [54]
Anti-scarring	Inhibit TGF-*β* receptor [27]

**Table 2:** Current study of EGCG incorporated biomaterials.

References	Types of Biomaterial	Composition	Study Design	Result	Conclusion
Zhao et al., 2021 [70]	Hydrogel	3-acrylamido phenylboronic acid (APBA), acrylamide	ROS Assay In vivo (diabetic induce)	ROS assay: Intracellular ROS reduction Wound: (Day 18) 2.0% wound closure, thicker tissue granulation	Able to induce intracellular ROS. In vivo: Promote tissue granulation, neovascularisation, angiogenesis, and high collagen deposition
Sun et al., 2020 [53]	Nanoparticles	Ascorbic acid, gelatine, chitosan	Immunohistochemical (IHC) staining Histological evaluation In vivo (animal study)	Wound: (Day 10) Epithelium completely healed in treatment group (visible neovascular vessels, hair follicles, and skin attachments) Healing rate: Significantly higher than other groups (*p* > 0.001).	In vivo: Accelerate collagen accumulation, enhance angiogenesis, and suppress inflammatory cells infiltration
S. Lee et al., 2020 [51]	Coated film	Polycaprolactone (PCL)	Ferric reducing antioxidant power (FRAP) assay ABTS radical scavenging assay TUNEL assay H_2_O_2_ scavenging assay	Apoptotic gene: ↓BAX Anti-apoptotic genes: ↑BCL2, ↑BCL2-L1 Anti-oxidative catalase: ↑FOXO3, ↑GPX-1	Effectively protect against ROS-induced oxidative damage
Kar et al., 2019 [52]	Hydrogel-based wound patches	Guar gum (GG), sodium alginate (SA), silver nanoparticles (AgNP)	In vivo (animal study)	Wound: (Day 9) ~30% remaining to heal (*p* < 0.0001) compared to control 75%	In vivo: Accelerate collagen deposition, wound healing, angiogenesis, neovascularisation, modulate growth factors, and inflammatory cytokines
Pires et al., 2019 [54]	Nanofiber membrane	Polycaprolactone (PCL), gelatine	H_2_O_2_ scavenging assay ROS induced UV radiation	H_2_O_2_ exposure: Membranes drops to ~20% compared to control ~45% UV exposure: Cell survival percentage > 76.9% (treatment); 52.9% (control)	Stagnant oxidative-stress cell damage
Liu et al., 2017 [58]	Hydrogel	Hyaluronic acid (HA)	ROS scavenging	Inhibit macrophages-mediated ROS	ROS levels significantly reduced compared to unstimulated macrophages
Chu et al., 2017 [56]	Membrane	Collagen	Cell viability and cell adhesion	0.064% collagen-EGCG shows highest cell proliferation	Downregulating MAPK signalling pathway, induce ROS

**Table 3:** Current study of EGCG incorporated treatment for wound healing.

References	Types of Treatment	Composition	Study Design	Result	Conclusion
Huang et al., 2019 [55]	Topical	Carboxymethylcellulose (CMC)	Immunohistochemical staining Molecular interaction assay	Wound: (Day 8) Better re-epithelialisation granulation and collagen deposition compared to control	In vitro: Inhibit overexpression of inflammatory cytokines and overactivated Notch signalling In vivo: Improved by inhibiting Notch signalling
Prakoeswa et al., 2020 [56]	Ointment	Hydrocarbon-based Eucerin	Clinical study	Wound: 84.11% size reduction, 85.45% depth reduction in the treatment group	In vivo: EGCG group (63.6%) Control group (30.8%) healed wound

## Data Availability

The data presented in this study are available on request from the corresponding author.
